# Associations between Prepartum and Postpartum Maternal Symptoms of Depression, Anxiety and Stress Related to COVID-19 Pandemic and Childhood Sensory Avoidance: Results from Conception Study

**DOI:** 10.3390/children11030352

**Published:** 2024-03-16

**Authors:** Delphine Aubin, Jessica Gorgui, Anick Bérard, Sarah Lippé

**Affiliations:** 1 Department of Psychology, University of Montreal, Marie-Victorin Building, 90 Vincent-D’Indy Avenue, Montreal, QC H2V 2S9, Canada; delphine.aubin@umontreal.ca; 2 CHU Sainte-Justine Research Center, University of Montreal, 3175 Chemin de la Côte-Sainte-Catherine, Montreal, QC H3T 1C5, Canada; jessica.gorgui@umontreal.ca (J.G.); anick.berard@umontreal.ca (A.B.); 3 Faculty of Pharmacy, University of Montreal, Jean-Coutu Building, 2940 Chemin de Polytechnique, Montreal, QC H3T 1J4, Canada; 4 Faculty of Medicine, Université Claude Bernard, Lyon 1, 69003 Lyon, France

**Keywords:** depression, anxiety, stress, prepartum, postpartum, sensory avoidance, COVID-19 pandemic

## Abstract

Background: Prepartum and postpartum maternal symptoms of stress, anxiety and depression are likely to influence the child’s sensory processing through hormonal alterations and an influence on mother–child interactions. Objective: We investigated the associations between maternal prepartum and postpartum symptoms of depression, anxiety and stress related to the COVID-19 pandemic and childhood sensory avoidance at 18 months. Methods: Longitudinal data from 409 participants followed during the COVID-19 pandemic were used. They completed questionnaires during pregnancy and up to 18 months after delivery. Maternal distress symptoms were assessed prenatally and at 18 months postnatally using the Edinburgh Postnatal Depression Scale, the Generalized Anxiety Disorders 7-item Scale and a 10-point scale assessing the level of stress felt related to the COVID-19 pandemic. Child sensory avoidance was assessed at 18 months postpartum using the Infant/Toddler Sensory Profile—Second Edition. Pearson correlations and multiple regressions measured the associations between maternal distress symptoms and child sensory avoidance. Results: Prepartum and 18-month postpartum maternal depression and anxiety were significantly correlated with childhood sensory avoidance (*p* < 0.05). Together, these variables explained 7.18% (F = 2.12, *p* < 0.05) of the variance of childhood sensory avoidance. Conclusions: These results support the contributory effect of prepartum and postpartum maternal distress on childhood sensory development.

## 1. Introduction

Pregnancy and the first few months following childbirth represent a period of mental-health vulnerability for women, as symptoms of depression, anxiety and stress are prevalent [1,2,3]. The COVID-19 pandemic has added further stressors, making everyone, but particularly women in the perinatal period, particularly vulnerable in terms of their well-being and mental health. During the pandemic, perinatal care was modified to comply with sanitary measures, social support was diminished due to social distancing measures and women could have additional worries about their health and the health of their child [4].

Child development is likely to be influenced by exposure to maternal prepartum and postpartum mental health. Exposure to prepartum and postpartum maternal stress has been associated with lower cognitive development [5,6], as well as to the development of a difficult temperament and the child’s social–emotional development [7,8]. Exposure to increased prepartum and postpartum maternal anxiety is associated with a risk of unfavourable development in the child’s social-emotional behaviour [9]. Exposure to maternal postpartum depression has been linked to the child’s cognitive [10] and behavioural [11,12] development.

Several aspects of the mother–child interaction may have consequences on child development [10,11,12]. Indeed, mothers experiencing depressive and anxiety-related symptoms may be less sensitive and attentive to their child’s needs, which can in turn prevent the child from developing a secure attachment, and lead to associated difficulties in social functioning and emotional regulation [11]. The lack of maternal sensitivity and attention may also affect the child’s temperament, influencing their behavioural development [12]. Moreover, depressed mothers may be less disposed to offer a sufficient stimulation [10]. Lack of stimulation may affect the quantity and quality of verbal interactions, affecting children’s cognitive development [10].

While most studies have focused on diminished environmental opportunities for children raised by mothers with distress symptoms, few have investigated the impact of maternal prepartum and postpartum mental health on the child’s ability to process sensory stimuli [13,14,15,16]. While sensory sensitivity varies from person to person, the way one perceives and responds to a stimulus can vary greatly [17]. Some people with sensory hypersensitivity will exhibit over-reactivity to sensory stimuli, which can lead to intense and aversive reactions [17]. On a developmental level, poor sensory processing skills in children are likely to have a negative impact on their social, cognitive and sensorimotor development [18]. According to the conceptual model presented by Dunn (1997), sensory avoidance corresponds to a sensory processing profile for which the neurological threshold for perception is low, i.e., there is sensory hypersensitivity, for which active self-regulatory strategies are employed to avoid excessive sensory stimulation [18]. Thus, children with sensory-avoidance behaviours may be reticent or withdraw from certain activities and adopt routines or rituals so that sensory input and neural activity become predictable and familiar patterns [18]. From a clinical perspective, these children may not be able to tolerate physical touch or commonly encountered sounds, have a restricted diet and withdraw from activities normally perceived as pleasurable, thus becoming overwhelmed by sensory stimulation, resulting in strong emotional reactions or disruptive behaviour [19].

Very few studies have investigated the links between exposure to symptoms of prepartum maternal distress and childhood sensory development [13,14,15,16], and even fewer on the biological mechanisms underlying these associations [14,15,16,20]. However, several studies have delved into the neurobiological and endocrine mechanisms that underly the association between prenatal maternal stress and foetal brain development. According to the foetal programming hypothesis, during critical periods of prepartum development, organ structure and function can be permanently altered as a function of the intrauterine environment and the time of exposure [21,22,23]. Prepartum stress is likely to reduce the activity of the 11β-hydroxysteroid dehydrogenase type 2 (11β-HSD2) enzyme, which regulates foetal exposure to cortisol by converting it into its inactive form, cortisone [21,24,25,26,27]. This could lead to increased foetal exposure to maternal cortisol, which may affect the neuroendocrine, structural and behavioural phenotype of the offspring [21,26,27]. More specifically, glucocorticoids have important effects on brain development, for instance by modulating synaptic development, as well as cell proliferation and differentiation in various brain regions [23,24,25]. Under high-stress conditions, long-term effects on foetal brain development could result from exposure to higher levels of maternal cortisol [27]. Downregulation of the placental 11β-HSD2 gene has also been associated with prepartum maternal anxiety and, to a lesser extent, prepartum maternal depression [28]. High levels of maternal stress could also lead to increased levels of corticotropin-releasing hormone (CRH) in the placenta, which, by activating CRH receptors, could influence the development of certain foetal brain areas rich in CRH receptors, like the amygdala, the hippocampus and limbic cortical areas [21,29]. For example, prepartum exposure to glucocorticoids leads to higher levels of CRH in the amygdala, which is associated with the altered functioning of this brain structure involved in the expression of anxiety and fear [25]. Prepartum maternal stress has also been associated with sleep disorders during pregnancy and a less healthy diet, which can lead to immune and inflammatory responses, as well as to nutrient deficiencies that can impair foetal brain development [22,24]. One theory to explain greater sensory sensitivity, and even sensory hypersensitivity, puts forward an imbalance between neuronal excitation and inhibition through differences in levels of the excitatory neurotransmitter glutamate or inhibitory neurotransmitter GABA (gamma-aminobutyric acid) in neural circuits of the neocortex involved in sensory perception [17,20]. Exposure to prepartum maternal stress is likely to disrupt the development of GABAergic and glutamatergic pathways in offspring, as it has been associated with the altered expression of certain receptor subunits and markers of synthesis and the transport of the GABAergic and glutamatergic pathways in the offspring [30,31].

Current evidence and recent studies highlight the impact of distress-provoking factors in women during the prepartum and postpartum period in association with the child’s neurological, cognitive and behavioural development [5,6,7,8,9,10,17,21,26,30,31,32,33]. However, few studies have focused on the potential effects of prepartum and postpartum maternal depression, anxiety and stress on children’s sensory development and behavioural responses during sensory stimulation [13,14,15,16,20]. There are still several factors and mechanisms related to childhood sensory avoidance that are not fully understood. Therefore, the aim of the present study is to assess the associations between prepartum and postpartum maternal symptoms of depression, anxiety and stress related to the COVID-19 pandemic and childhood sensory avoidance.

## 2. Materials and Methods

### 2.1. Data and Procedures

The data used in this correlational study come from the CONCEPTION longitudinal study [5], in which women in the perinatal period during the COVID-19 pandemic were followed from pregnancy to two years after delivery. This study aimed to assess how maternal mental health was affected by the COVID-19 pandemic and its associated policies. Data were also collected on the delivery and development of their child born during the COVID-19 pandemic. Participants completed online questionnaires at three time points. The first set of questionnaires was completed in the prepartum period, regardless of the participant’s trimester of pregnancy. Participants also completed questionnaires at 2 months and 18 months postpartum. Consent and data were collected online via SurveyMonkey^®^’s secure survey platform. Women were recruited via press releases and mainstream media interviews (i.e., UdeMNouvelles, CTV News Chanel, University of Manitoba News, Montreal Gazette and Naître et Grandir), who relayed advertisements and preliminary results of the Conception study, and via social media (Facebook, Instagram, Twitter, LinkedIn and TikTok) through an account created for the Conception study, from which members of the Conception team promoted the study. Direct recruitment also took place at community associations for new immigrants in Montreal (Canada) and via QR codes on posters in OB/GYN clinics in Quebec province (Canada), providing direct access to the questionnaires with a mobile device. Women were able to complete the questionnaires in French, English, Mandarin, Spanish or Portuguese. All collected data are centralized at CHU Ste-Justine in Montreal, QC, Canada.

### 2.2. Sample

This study used data from 409 pregnant participants recruited between 23 June 2020 and 15 July 2021 as part of the CONCEPTION longitudinal study. Participants included in this study (i) were pregnant at the time of recruitment, (ii) had to be at least 18 years old and (iii) had to have reached the 18-month-postpartum measurement time. For the first time point (prepartum), 368 participants (89.7%) fully completed their questionnaires, 39 participants (9.53%) partially completed their questionnaires, and 2 participants (0.48%) did not complete their questionnaires at all. For the second time point (2 months postpartum), 251 participants (61.36%) fully completed their questionnaires, 10 participants (2.44%) partially completed their questionnaires, and 148 participants (36.18%) did not complete their questionnaires at all. For the third time point (18 months postpartum), 333 participants (81.42%) fully completed their questionnaires, and 76 participants (18.58%) partially completed their questionnaires.

### 2.3. Measures

#### 2.3.1. Demographic and Physical- and Mental-Health Data

Data were prenatally collected on physical health, socio-demographic characteristics (age, ethnic background, education, household income, marital status and area of residence), pregnancy history (number of past pregnancies, abortions and miscarriages), medical diagnosis (asthma, nausea, high blood pressure, thyroid disease, diabetes, high cholesterol, migraines, cancer, epilepsy, hearth disease, lung disease, anaemia or blood disease, ulcer or stomach disease, kidney disease, liver disease, pain, influenza, infection or none), treatment (asthma, nausea, high blood pressure, thyroid disease, diabetes, high cholesterol, migraines, cancer, epilepsy, hearth disease, lung disease, anaemia or blood disease, ulcer or stomach disease, kidney disease, liver disease, pain, influenza, infection or none), health-related behaviours (i.e., smoking, alcohol consumption, drug use and exercise), health concerns and the use of prescribed medication, as well as participants’ employment status and any changes since the start of the pandemic. 

Participants also completed scales assessing their well-being and mental health using validated tools. These scales were completed prenatally, as well as at 2 months and at 18 months postnatally. Maternal depression was measured using the Edinburgh Postnatal Depression Scale (EPDS) [34]. The EPDS is a questionnaire assessing perinatal depressive and anxiety symptoms using 10 items rated on a 4-level Likert scale. This instrument has good internal consistency (total scale and subscales: Cronbach α ≥ 0.88), as well as satisfactory validity [34,35]. The Generalized Anxiety Disorder 7-item Scale (GAD-7) [36] was used to assess the presence and severity of generalized anxiety disorders. With a 4-point Likert scale, the GAD-7 has excellent internal consistency (Cronbach α = 0.92), good test–retest reliability (intraclass coefficient (ICC) = 0.83), and good factorial and content validity [36]. The level of stress related to the COVID-19 pandemic was measured using a visual analogue scale ranging from 0 (no stress) to 10 (extreme stress), on which participants were asked to indicate their level of overall stress experienced since the beginning of the COVID-19 pandemic. 

#### 2.3.2. Child Health, Development and Sensory Avoidance

Data were collected at 2 months and 18 months postpartum on child health (sex, weight, gestational age at birth, neonatal events, COVID-19 infection, febrile seizures, major malformation and genetic diagnoses, medical complications and hospitalization) and sleep patterns. Aspects of child development progress were assessed at 18 months postpartum using Ages and Stages, Third Edition (ASQ-3), which is an assessment tool used for evaluating children’s developmental progress in five developmental areas (i.e., communication, gross motor, fine motor, problem solving and personal–social) [37]. The ASQ-3 is used to assess whether the child’s development appears to be on time, should be monitored or is at risk of developmental delays [37]. The ASQ-3 has good test–retest reliability (intra-class coefficients (ICC) = 0.75–0.82), poor to good internal consistency (Cronbach’s Alpha = 0.51–0.87), according to developmental domains and age intervals, and good predictive and discriminant validity [38].

Childhood sensory avoidance was measured at 18 months postpartum using the Avoidance subscale of the Infant/Toddler Sensory Profile—Second Edition [39], which is a 54-item questionnaire completed by the caregiver to assess how the child responds to sensory events in daily life. The caregiver is asked to indicate the frequency with which the child performs the described behaviour on a 5-point Likert scale: almost always (90% of the time or more), frequently (75% of the time), half the time (50% of the time), sometimes (25% of the time) or almost never (10% of the time or less). The questionnaire is divided into six sections. The first section covers the general treatment, which assesses the child’s responses to routines and schedules. The next four sections cover auditory, visual, tactile, vestibular and oral sensory processing. The final section covers behavioural responses related to sensory-information processing. The questions in these sections are coded according to the quadrant of sensory tendencies they represent (i.e., Sensation Seeking, Registration, Sensory Sensitivity and Sensory Avoidance). For example, the item “resists being cuddled” is part of the tactile-information-processing section, and the item “shows a clear aversion to almost all foods” is part of the oral sensory-information-processing section, but both items are also part of the quadrant corresponding to sensory avoidance. Total raw scores for the quadrants, including the sensory avoidance quadrant, are calculated by summing the raw scores of the items in the questionnaire corresponding to the quadrant. The Infant/Toddler Sensory Profile—Second Edition has poor to good internal consistency (Cronbach’s alpha = 0.57–0.80), good to excellent test–retest reliability (intra-class coefficients (ICC) = 0.83–0.92) and good content and construct validity based on the Quality Criteria for Health Status Questionnaires (QCHSQ) [40,41]. The Sensory-Avoidance subscale is thus a fair summary of sensory-processing anomalies. 

### 2.4. Statistical Analyses

Statistical analyses were carried out using RStudio (version 2023.06.1+524, Posit Software, PBC, Boston, MA, USA). Preliminary analyses were carried out to perform descriptive analyses and quantify the percentage of missing data for maternal prepartum and 2- and 18-month postpartum depression, anxiety and stress related to the COVID-19 pandemic and childhood sensory avoidance at 18 months. Raw scores from questionnaires (EPDS, GAD-7 and Sensory Profile) were used to create continuous variables. The stress level related to the COVID-19 pandemic, assessed using a 10-point Likert scale question, was considered as a continuous variable. In order to control for known variables likely to affect prepartum and postpartum maternal distress (i.e., main occupation, cultural group, marital status, income, education and number of past pregnancies/abortions/miscarriages) or child development (i.e., trimester of pregnancy, gestational age at recruitment in weeks, the woman’s medical diagnosis, the woman’s medical treatment, substance use during pregnancy [alcohol, cigarettes, drugs and cannabis], and the infant’s medical condition), their relation was tested against all maternal distress symptoms and childhood sensory avoidance in preliminary analyses. The relation between certain aspects of child development (communication, gross motor, fine motor, problem solving, social–relational) and maternal distress symptoms or childhood sensory avoidance was also tested using Ages and Stages (ASQ-3). Only variables statistically correlated with prepartum and postpartum depression, anxiety and stress related to the COVID-19 pandemic, or childhood sensory avoidance, were included as control variables in the statistical analyses. Pearson correlations were performed to determine the associations between self-reported maternal symptoms of depression, anxiety and stress related to the COVID-19 pandemic at each time point (prepartum, 2- and 18-months postpartum) and childhood sensory avoidance measured at 18 months. Correlations were corrected for multiple comparisons using the Holm–Bonferroni adjustment, which is more powerful than the Bonferroni adjustment [42,43]. Then, a multiple regression with all statistically significant variables was performed to determine whether emotional symptoms reported by the mother (depression, anxiety and COVID-19-related stress) predict childhood sensory avoidance at 18 months.

### 2.5. Ethics

The CHU Sainte-Justine’s Research Ethics Committee has approved the study (no. MP-21–2021-2973).

## 3. Results

### 3.1. Participants

The majority of participants lived in Canada (94.38%), particularly in the province of Quebec (85.09%), identified as white (92.67%) and lived in urban cities (45.97%) or suburbs (42.30%) (Table 1). Socioeconomic status was relatively high, with only 1.47% of participants having incomes below the Canadian pre-tax low-income cut-off for people living in a metropolitan area [44]. The average number of years of schooling completed was 17.23 (*SD *= 4.72), which corresponds approximately to the time required to complete a bachelor’s degree.

At the time that prepartum questionnaires were completed, participants’ mean gestational age was 18.7 weeks. The majority of participants were in their second trimester of pregnancy (68.46%), and fewer were in their first trimester (23.96%) or third trimester (7.09%). The mean gestational age at birth was 39.2 weeks (*SD *= 1.60). Most participants had no previous deliveries (48.66%), abortions (77.02%) or miscarriages (63.81%) (Table 2). Nausea (17.36%), thyroid disease (10.02%), asthma (9.05%), migraines (5.62%) and high blood pressure (3.42%) were the most prevalent and treated diagnoses among pregnant participants. The majority of participants (45.23%) had no diagnoses and were not receiving medical treatment (Table 2). Few infants had medical conditions (Table 3), and the majority showed normal development in communication, gross-motor, fine-motor, problem-solving and interpersonal skills measured at 18 months (Table 4 and Table 5). For the ASQ, data from 342 children were obtained for the communication, gross-motor and fine-motor scales (missing data: 16.38%), and data from 336 children were obtained for the problem-solving and personal–social scales (missing data: 17.85%).

### 3.2. Preliminary Analyses

As marital status, trimester of pregnancy, gestational age at recruitment in weeks, household income, cigarette smoking during pregnancy and whether or not it is a first pregnancy, as well as number of past deliveries, abortions and miscarriages were statistically correlated with prepartum and postpartum depression, anxiety and stress related to the COVID-19 pandemic, or sensory avoidance (*p* < 0.05), they were included in all statistical analyses as control variables. As certain diagnoses (i.e., asthma, high blood pressure, migraines, anaemia or blood disease, diabetes, ulcer or stomach disease) and treatments (i.e., asthma, anaemia or blood disease) were associated with prepartum and 18-month postpartum depression, anxiety and stress related to the COVID-19 pandemic (Table 6), the dichotomous variables “presence of a diagnosis” (yes, no) and “presence of a treatment” (yes, no) were created and integrated into all statistical analyses. All variables were normally distributed, while skewness and kurtosis indices were all +/−2 and +/−7, respectively [45]. All extreme scores (+/−3.29 *SD*) were identified and replaced by scores at the limit of z = +/−3.29 using the winsorizing method [46]. Since between 37.9% and 38.63% of data were not provided by participants for depression, anxiety and stress related to the COVID-19 pandemic at 2 months postpartum, this measurement time was removed from all statistical analyses. For prepartum and 18-month postpartum depression and stress related to the COVID-19 pandemic, as well as for 18-month postpartum anxiety, the percentage of missing data ranged from 2.2% to 4.9%. Given the higher percentage of missing data for prepartum anxiety (9.5%) and sensory avoidance at 18 months (18.6%; data were available for 333 children), all statistical analyses were performed using multiple imputations, as it is a recommended method for generating unbiased estimates when the number of missing data is moderate (i.e., ≤20% for data missing at random) [47]. Data were assumed to be missing at random (MAR), as there were no demographic characteristics or trends in the available data for these participants to suggest that the missing data were not random, and that Little’s MCAR test, which was statistically significant (*p* < 0.001), suggested that data were not missing completely at random. The Fully Conditional Specification algorithm (FSC) [38,48] was used to create 20 imputed databanks [49] from maternal prepartum and 18-month postpartum depression, anxiety and stress related to the COVID-19 pandemic, childhood sensory avoidance at 18 months and control variables.

### 3.3. Associations between Maternal Symptoms and Sensory Avoidance

**Figure 1:**
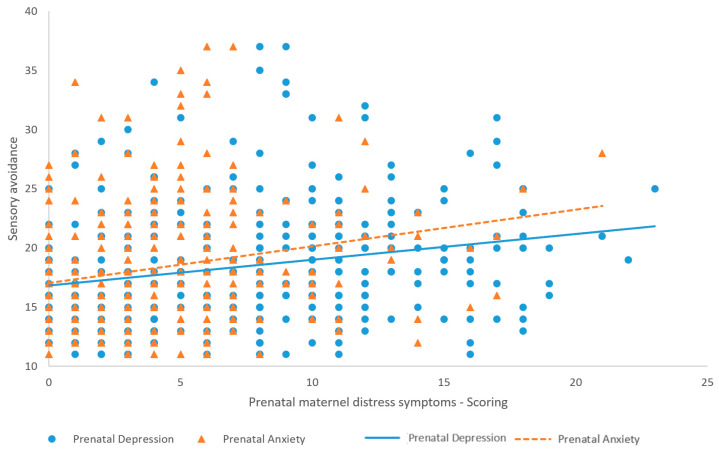
Correlations and regression lines between maternal prepartum anxiety and depression and childhood sensory avoidance.

**Figure 2:**
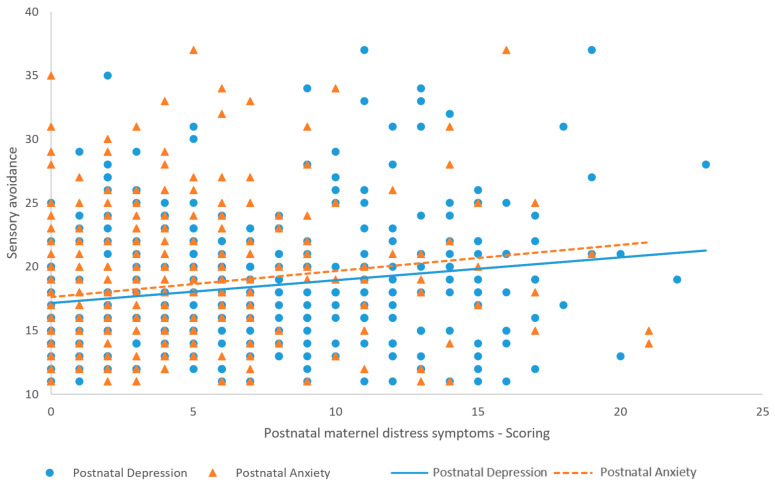
Correlations and regression lines between maternal postpartum anxiety and depression and childhood sensory avoidance.

In the prepartum period, maternal depression (r(407) = 0.19, *p* = 0.0004) and anxiety (r(407) = 0.18, *p* = 0.0006) were significantly correlated with childhood sensory avoidance at 18 months (Figure 1), but not maternal stress related to the COVID-19 pandemic (r(407) = 0.09, *p* = 0.06). Maternal depression (r(407) = 0.17, *p* = 0.0008) and anxiety (r(407) = 0.16, *p* = 0.002) at 18 months postpartum were significantly correlated with childhood sensory avoidance at 18 months (Figure 2), but not maternal stress related to the COVID-19 pandemic at 18 months postpartum (r(407) = 0.08, *p* = 0.15). The strength of the associations indicates that prepartum and 18-month postpartum depression and anxiety are weakly associated with childhood sensory avoidance at 18 months [50]. All significant correlations were positive, meaning that a higher level of prepartum and 18-month postpartum depression and anxiety is associated with a higher level of childhood sensory avoidance at 18 months.

The variables significantly correlated with childhood sensory avoidance at 18 months (i.e., prepartum and 18-month postpartum maternal depression and anxiety) and control variables were entered into a multiple regression as independent variables. Together, these variables significantly explained 7.18% of the variance of childhood sensory avoidance at 18 months (F(10, 398) = 2.12, *p* < 0.05, R^2^ = 0.07). Within this model, none of the maternal symptoms (i.e., prepartum and 18-month postpartum depression and anxiety) was significantly associated with childhood sensory avoidance at 18 months (Table 7).

## 4. Discussion

This study demonstrates that higher levels of maternal prepartum and 18-month postpartum symptoms of depression and anxiety are associated with higher levels of childhood sensory avoidance at 18 months. The results support that together, prepartum and 18-month postpartum depression and anxiety predict the variance of childhood sensory avoidance at 18 months. However, none of these maternal distress symptoms stand out as a better predictor of childhood sensory avoidance at 18 months. These results concur with those of Gee et al. (2021) [13], who found that higher levels of maternal prepartum anxiety and depression, as well as higher levels of maternal anxiety and depression at 6 months postpartum, were associated with more “outside of the majority” scores on the sensory sensitivity scale in 14-month-old infants. Whereas sensory avoidance involves active self-regulatory strategies to sensory overstimulation, sensory sensitivity is characterized by passive responses to sensory overstimulation [18]. Our results, together with those obtained by Gee et al. (2021) [13], suggest an association between prepartum and postpartum maternal distress and sensory processing profiles characterized by sensory hypersensitivity. Hence, our study complements the latter, indicating that the association is maintained in time, and suggesting the association between maternal prepartum and postpartum distress symptoms and infants’ sensory sensitivity symptoms can also involve avoidance behaviours.

Associations found between maternal postpartum depression and anxiety and childhood sensory avoidance suggest that maternal postpartum distress might influence children’s behavioural responses to sensory overstimulation. Indeed, postpartum maternal distress is susceptible to influencing childhood behavioural development through a reduced number of positive mother–infant interactions [12]. In particular, maternal postpartum distress has been associated with a child’s difficult temperament [8], which is reflected in withdrawal responses to new sensorial stimulation and intense emotional responses [51]. These responses associated with having a difficult temperament are in line with active withdrawal and avoidance behaviours observed in children who are prone to sensory avoidance [18].

Since maternal prepartum depression and anxiety symptoms were also associated with child sensory avoidance at 18 months, the involvement of biological mechanisms in these associations are likely. It was suggested that prepartum maternal stress could increase foetal exposure to maternal cortisol and increased levels of corticotropin-releasing hormone (CRH) in the placenta [21,28]. This could affect the neuroendocrine, structural and behavioural phenotype of the offspring, as well as the development of certain foetal-brain areas rich in CRH receptors [21,26]. Maternal prepartum stress has also been linked to the alteration of the expression of certain components of the GABAergic and glutamatergic pathways [30,31]. An imbalance in the levels of the excitatory neurotransmitter glutamate or the inhibitory neurotransmitter GABA is likely to lead to hyperexcitability in the neocortex neural circuits involved in sensory perception [17,20,52]. Prepartum maternal stress has also been associated with alterations in the serotonergic and norepinephrine systems, with neurotransmitters playing a crucial role in brain development [53,54,55,56,57]. Several brain-development processes (cell migration, neuronal division and synaptogenesis) are influenced by serotonin, which acts as a trophic factor [58]. The development of noradrenergic neurons is regulated by norepinephrine, which contributes to the shaping of the nervous system [56]. The disruption of these processes by excessive or insufficient levels of these neurotransmitters is likely to alter the developmental trajectory of the brain, leading to lasting changes in brain function [54,56,58]. In fact, prepartum exposure to selective serotonin reuptake inhibitors (SSRIs) has been associated with anatomical and functional changes in somatosensory cortical neurons [55]. For instance, SSRI use during pregnancy was associated with improved infant performance on a language discrimination task, suggesting accelerated perceptual development in the auditory cortex [15]. Consequently, exposure to maternal prepartum stress could affect the development of certain brain areas involved in cerebral excitability and sensory processing, lowering the neurological threshold [17,20,21,26,52,53,54,55,56,57,58,59,60].

Genetic factors could also partly explain the results obtained in our study. Sensory-processing abnormalities are particularly common in children and adults with neurodevelopmental disorders caused by genetically guided mechanisms, such as autism spectrum disorders (ASDs) [61]. Studies have shown a greater prevalence of sensory abnormalities among parents of children with ASDs than among parents of typically developing children [61,62], and among parents of multiplex ASD families than among parents of simplex ASD families [61]. The concordance rate of defensive behaviours to tactile and auditory stimuli was also higher in monozygotic than in dizygotic twins [63]. Some polymorphisms in the CNTNAP2 (contactin-associated protein-like 2) and Gabrb3 genes, which are known genetic risk factors for ASD, were associated with hypersensitivity to heat and tactile stimuli [64,65,66]. These findings support the idea that genetic predispositions could also lead to sensory-processing abnormalities and could partly explain the results obtained in our study.

Our results could also be explained by epigenetic modifications induced by prepartum and postpartum exposure to maternal stress. Indeed, according to the Dual-Activation Hypothesis, exposure to prepartum maternal stress during critical periods of neural development may lead, via hormonal and synaptic alterations, to epigenetic modifications in stress-related and other connected neural networks, including sensory and emotional networks [14]. Genes coding for the glucocorticoid receptor (Gr) [14,67,68,69,70] and the FK506-binding protein 5 (Fkbp5) [14,67] in the hippocampus, along with the neuropeptide arginine vasopressin (Avp) [14,67,68,71] and the corticotropin-releasing factor (Crf) [14,67] in the hypothalamus, may be implicated in the epigenetic programming of the stress response [14]. These epigenetic modifications, while facilitating specific adaptations to stressors, may also lead to functional maladaptation depending on the timing and the intensity of the stimulation [14,72,73]. For example, prior research has shown alterations in brain structures, such as increased grey matter density in the left superior temporal gyrus of the primary auditory cortex in children exposed to verbal abuse [14,72], and reduced grey matter volume in the visual cortex in those witnessing domestic violence [14,73]. Thus, the associations we observed between prepartum and postpartum maternal depression and anxiety could also result from epigenetic changes due to early exposure to maternal distress. To acquire a thorough understanding of the transgenerational transmission of sensory sensitivity, it is essential to gather genetic, epigenetic and symptom evaluations from both infants and parents.

Moreover, our study stands out for having examined prepartum and postpartum maternal stress related to the COVID-19 pandemic and its possible association with sensory avoidance. However, prepartum and 18-month postpartum stress related to the COVID-19 pandemic were not associated with or predictive of infant sensory sensitivity. This was not expected, considering the literature supporting a link between prepartum and postpartum maternal stress and child development [6,8,21,26]. Indeed, it is possible that stress specifically linked to the COVID-19 pandemic has not had a detectable effect on child development, but overall maternal stress does. In fact, our results suggest that regardless of the reason or source of the distress experienced by the mother, it can influence the child’s sensory development. On the other hand, our sample was made up of participants who were predominantly employed, with high incomes and living with a partner. Therefore, our participants may had protective factors that prevented them from being impacted by the additional stress factors arising from the pandemic (e.g., social isolation, job loss and lockdown) [4,74]. Also, the level of distress associated with the COVID-19 pandemic could have fluctuated considerably depending on the level of contagion and public health policies in place, which were modified considerably throughout the pandemic. The level of distress could also have stabilized or decreased over time as the COVID-19 pandemic unfolded over a long period of time. Our scale, which assessed the overall stress levels experienced in relation to the COVID-19 pandemic, may thus have lacked sensitivity. Future studies could evaluate the effects of prepartum and postpartum maternal distress experienced during the COVID-19 pandemic while considering the different waves of the pandemic, as well as the impacts of certain additional stressors resulting from the pandemic (e.g., job loss and social isolation).

Most participants were in their second trimester of pregnancy when they completed their prepartum questionnaires, which is a particularly vulnerable window for foetal neurodevelopment, as neurogenesis and neuronal migration processes are particularly active [75,76]. Thus, exposure to prepartum maternal stress during the second trimester of pregnancy may particularly influence foetal brain development [75,76,77], affecting subsequent childhood cognitive development [78]. As we found a higher effect of anxiety during the prepartum period, and considering that most of our participants were in their second trimester when they completed their prepartum questionnaires, our results corroborate studies regarding the timing of exposure to prepartum maternal distress on child development [75,76,77,78]. On the other hand, foetal brain development begins as early as the first trimester of pregnancy. As early as the fifth week of pregnancy, all major subdivisions of the brain (i.e., myelencephalon, metencephalon, mesencephalon, diencephalon and telencephalon), as well as the interhemispheric fissure, are identifiable [79]. The thickening of the hippocampus begins at 5 weeks postmenstrual age (PMA) [79]. Moreover, maternal distress experienced during the first trimester of pregnancy has been associated with the child’s cognitive development [78] and having difficult temperament [80,81]. While a proportion of our participants were in their first trimester of pregnancy at the time of completing their prepartum questionnaires, distress experienced during this trimester of pregnancy may also have influenced the results obtained in this study.

Associations were found between some maternal diagnoses (i.e., asthma, high blood pressure, migraines, anaemia or blood disease, diabetes, ulcer or stomach disease) and treatments (i.e., asthma, anaemia or blood disease), and maternal prepartum and postpartum depression, anxiety and stress related to COVID-19. These findings are consistent with studies investigating the distress experienced by pregnant women with chronic illnesses [82,83,84]. Some chronic illnesses (e.g., diabetes, thyroid and kidney disease) can lead to adverse outcomes for pregnant women (e.g., pre-eclampsia and gestational hypertension) [83,85,86,87] and their infants [83,88,89]. Pregnant women with chronic illnesses may therefore experience distress resulting from managing the risk of these chronic diseases on their health and that of their infant [83], or from perceptions of a lack of control and low self-efficacy [82]. Interestingly, even after controlling for the potential impacts of chronic diseases during pregnancy on foetal development and maternal stress [82,83,84,85,86,87,88,89], associations were still observed between prepartum and 18-month postpartum maternal depression and anxiety and childhood sensory avoidance.

The respective associations between prepartum and 18-month postpartum symptoms of maternal distress and child sensory avoidance at 18 months were observed among children with typical development. In this sample of the CONCEPTION study, the developmental spheres assessed using the ASQ-3 (i.e., communication, gross motor, fine motor, problem solving and personal–social) were not significantly associated with prepartum and 18-month postpartum symptoms of depression, anxiety and stress related to the COVID-19 pandemic, nor with sensory avoidance at 18 months. However, numerous studies support an association between exposure to prepartum and postpartum symptoms of maternal distress and childhood cognitive, motor, behavioural and socio-emotional development [6,8,9,10,11,12]. Given that a large proportion of the children in the sample showed normal development across the different developmental spheres assessed (i.e., between 60.8% and 72.6%), it is possible that prepartum and 18-month postpartum maternal distress did not impact the developmental spheres as assessed through the ASQ-3 for the majority of children. Thus, the associations observed between prepartum and 18-month postpartum symptoms of maternal distress and childhood sensory avoidance at 18 months were unlikely to be influenced by atypical child development.

The limitations of this study must be considered. For example, some of the measurement scales used are very circumscribed in time, since the depression- and anxiety-screening scales assess symptoms experienced in the seven to fourteen days preceding the completion of the questionnaire. Thus, it is not possible to know whether these symptoms persisted over time, and what effect the persistence of symptoms would have had on the study results. However, we have used validated tools to measure maternal mental health, which yields results indicative of symptoms of depression and anxiety. Also, this study cannot reveal the relation between sensory avoidance and temperament peculiarities, which have been observed as influencing child behavioural development [90]. This limits the interpretation of the observed associations between maternal distress symptoms and sensory avoidance, which could be due either to neurological reasons, such as a particular sensory-processing profile such as sensory hypersensitivity, or to behavioural tendencies, arising, for example, from a difficult temperament. In addition, since our sample consisted mainly of white Canadians with a high socioeconomic status, the generalizability of our results to diverse populations is limited. To ensure better generalizability, future studies investigating the associations between maternal mental health and childhood sensory development should include participants from more diverse cultural communities and socioeconomic statuses. Lastly, all variables were measured from questionnaires completed by the mothers, including variables relating to their child. The condition of the mother could have led to a bias in the evaluation of variables related to the child’s health and development. The size of the observed associations could have been artificially inflated. Despite these limitations, this study revealed for the first time associations between prepartum maternal emotional symptoms and sensory avoidance, which can subsequently affect infants’ and children’s behaviours. As a result, the findings reveal new mechanistic avenues to explain the associations between prepartum maternal emotional symptoms and postpartum childhood neurodevelopment.

## 5. Conclusions

From a sample of women in the perinatal period during the COVID-19 pandemic, our study has shown that maternal prepartum and 18-month postpartum symptoms of depression and anxiety are associated with childhood sensory avoidance at 18 months. Together, prepartum and 18-month postpartum depression and anxiety predict the variance of childhood sensory avoidance at 18 months. The observed associations are correlational. Although the influence of maternal mental health on the child’s sensory development is understudied, our study is in line with the literature supporting an association between exposure to prepartum and postpartum maternal distress and child cerebral and behavioural development. This study highlights the importance of considering the influence of maternal mental health on children’s sensory development in future studies and in clinical care.

## Figures and Tables

**Table 1:** Characteristics of participants.

	N = 409	%	Mean	SD	Min	Max
Country of residence						
Canada	386	94.38				
United States	9	2.20				
France	6	1.47				
Sweden	1	0.24				
Democratic Republic of Congo	2	0.49				
United Arab Emirates	1	0.24				
Not specified	4	0.98				
Province						
Quebec	348	85.09				
Ontario	25	6.11				
British Columbia	4	0.98				
Alberta	6	1.47				
Manitoba	2	0.49				
New Brunswick	1	0.24				
Nova Scotia	2	0.49				
Not specified	21	5.13				
Area of residence						
Urban	188	45.97				
Suburban	173	42.30				
Rural	44	10.76				
Not specified	4	0.98				
Ethnic background						
White	379	92.67				
Asian	9	2.20				
Black	7	1.71				
Hispanic	2	0.49				
Aboriginal (North American Indians, Metis or Inuit [Inuk])	2	0.49				
Other	7	1.71				
Not specified	3	0.73				
Years of schooling	404	98.77	17.23	4.72	0	32
Not specified	5	1.22				
Employment status						
Employed—Full time	303	74.08				
Employed—Part time	26	6.36				
On welfare	6	1.47				
Self-employed	35	8.56				
Student/Intern	15	3.67				
Unemployed	18	4.40				
Prefer not to answer	2	0.49				
Not specified	4	0.98				
Living situation						
Living with a partner/married	389	95.11				
Living alone or single mother	12	2.93				
Living with parents/family	4	0.98				
Other	1	0.24				
Prefer not to answer	1	0.24				
Not specified	2	0.49				
Household income, CAD						
<CAD 30,000	6	1.47				
CAD 30,000–CAD 60,000	31	7.58				
CAD 60,001–CAD 90,000	58	14.18				
CAD 90,001–CAD 120,000	108	26.41				
CAD 120,001–CAD 150,000	71	17.36				
CAD 150,001–CAD 180,000	52	12.71				
>CAD 180,000	60	14.67				
Prefer not to answer	20	4.89				
Not specified	3	0.73				

**Table 2:** Women’s pregnancy history.

	N = 409	%	Mean	SD	Min	Max
Age at recruitment in years	407	99.51	32.64	3.96	21.17	45.87
Not specified	2	0.48				
Gestational age at recruitment in weeks	407	99.51	18.68	6.48	5	38
Missing value	2	0.48				
Gestational age in weeks at birth	267	65.28	39.2	1.60	31	43
Missing value	142	34.72				
Trimester of pregnancy at the time of survey completion						
First trimester	98	23.96				
Second trimester	280	68.46				
Third trimester	29	7.09				
Not specified	2	0.49				
First Pregnancy						
Yes	164	40.1				
No	243	59.41				
Missing value	2	0.49				
Pregnancy type						
Singleton (one baby)	400	97.8				
Twins (two babies)	4	0.98				
Missing value	5	1.22				
Number of past deliveries						
Zero	199	48.66				
One	138	33.74				
Two	42	10.27				
Three	9	2.2				
Four	2	0.49				
Six	2	0.49				
Missing value	17	4.16				
Number of past abortions						
Zero	315	77.02				
One	40	9.78				
Two	11	2.69				
Four	1	0.24				
Missing value	42	10.27				
Number of past miscarriages						
Zero	261	63.81				
One	84	20.54				
Two	21	5.13				
Three	14	3.42				
Four	4	0.98				
Five	1	0.24				
Missing value	24	5.87				
Medical diagnosis						
Asthma	37	9.05				
Nausea	71	17.36				
High Blood Pressure	14	3.42				
Thyroid disease	41	10.02				
Diabetes	13	3.18				
Migraines	23	5.62				
Hearth Disease	5	1.22				
Lung Disease	1	0.24				
Anemia or other blood disease	2	0.49				
Ulcer/Stomach Disease	2	0.49				
Kidney disease	1	0.24				
Liver disease	2	0.49				
Pain	4	0.98				
Infection	7	1.71				
None	185	45.23				
Missing value	2	0.48				
Medical treatment						
Asthma	25	6.11				
Nausea	64	15.65				
High Blood Pressure	5	1.22				
Thyroid disease	37	9.05				
Diabetes	5	1.22				
Hearth Disease	1	0.24				
Anemia or other blood disease	20	4.89				
Ulcer/Stomach Disease	7	1.71				
Kidney disease	1	0.24				
Liver disease	1	0.24				
Pain	2	0.49				
Infection	6	1.47				
None	194	47.43				
Missing value	2	0.49				

**Table 3:** Children’s medical conditions.

	N = 409	%
Blind or partially blind	1	0.24
Nystagmus	1	0.24
Strabismus	2	0.49
Abnormal muscle tone/muscle weakness	4	0.98
Is gaining weight too slowly	30	7.33
Is gaining weight too quickly	3	0.73
Vitamin or mineral deficiencies	1	0.24
Difficulties being fed	2	0.49
Cardiac anomaly confirmed by cardiac ultrasound	4	0.98
Stridor	5	1.22
Apnea	1	0.24
Asthma	13	3.18
Stomach infection or frequent diarrhea	1	0.24
Syndrome or condition that affects heart development	1	0.24
Brain injury	1	0.24

Note: Medical conditions shown by at least one child from the sample.

**Table 4:** Child development: ages and stages at 18 months.

	N = 409	%
Communication		
Above the cut-off (30–60)	270	66.01
Close to the cut-off (15–29)	61	14.91
Below the cut-off (0–14)	11	2.68
Missing value	67	16.38
Gross motor		
Above the cut-off (46–60)	287	70.17
Close to the cut-off (36–45)	19	4.64
Below the cut-off (0–35)	36	8.8
Missing value	67	16.38
Fine motor		
Above the cut-off (45–60)	297	72.61
Close to the cut-off (35–44)	35	8.55
Below the cut-off (0–34)	10	2.44
Missing value	67	16.38
Problem solving		
Above the cut-off (36–60)	249	60.88
Close to the cut-off (26–35)	55	13.44
Below the cut-off (0–25)	32	7.82
Missing value	73	17.84
Personal–social		
Above the cut-off (38–60)	291	71.14
Close to the cut-off (27–37)	36	8.8
Below the cut-off (0–26)	9	2.2
Missing value	73	17.84

Note: Above the cut-off = child’s development appears to be on schedule; close to the cut-off = child is in the monitoring zone.

**Table 5:** Ages and stages at 18 months: measures of central tendency and dispersion.

	N	Mean	SD	Min	Max
Ages and stages at 18 months					
Communication	342	37.73	13.51	0	60
Gross Motor	342	52.98	11.32	13.03	60
Fine Motor	342	52.37	8.38	22.76	60
Problem Solving	336	43.94	11.48	6.13	60
Personal–Social	336	46.84	9.15	16.19	60

**Table 6:** Associations between maternal prepartum and postpartum depression, anxiety and stress related to the COVID-19 pandemic, and prepartum medical diagnosis and treatment.

	Prepartum Depression	Postpartum Depression	Prepartum Anxiety	Postpartum Anxiety	Prepartum Stress Related to COVID-19 Pandemic	Postpartum Stress Related to COVID-19 Pandemic
Medical diagnosis					−0.25 **	
Asthma			−0.24 *		−0.25 *	
High Blood Pressure	−0.33 **	−0.29 *	−0.33 **	−0.24 *	−0.23 *	
Diabetes				−0.24 *	−0.22 *	
Migraines	−0.21 *		−0.22 *			
Anemia or other blood disease			−0.24 *			
Ulcer/Stomach Disease					0.24 *	
Medical treatment						
Asthma	−0.2 *					
Anemia or other blood disease			−0.26 *			

* *p* < 0.05, ** *p* < 0.01.

**Table 7:** Associations between maternal prepartum and 18-month postpartum distress symptoms and childhood sensory avoidance within the multiple regression model.

	Estimate	Standard Error	*p* Value
Prepartum depression	0.08	0.08	0.31
18-month postpartum depression	0.08	0.07	0.25
Prepartum anxiety	0.10	0.1	0.30
18-month postpartum anxiety	0.04	0.09	0.62

## Data Availability

Anonymized individual-level data from the study including data dictionaries, data collection tools will be made available upon request. Requests for access will be reviewed by a data-access committee. The data are not publicly available due to the absence of authorization in consent forms allowing public publication of participants’ data.
